# Prevalence of Ectoparasitic Infections and Other Dermatological Infections and Their Associated Factors among School Children in Gampaha District, Sri Lanka

**DOI:** 10.1155/2019/5827124

**Published:** 2019-03-25

**Authors:** Nayana Gunathilaka, Nilmini Chandrasena, Lahiru Udayanga

**Affiliations:** ^1^Department of Parasitology, Faculty of Medicine, University of Kelaniya, Ragama, Sri Lanka; ^2^Department of Bio-systems Engineering, Faculty of Agriculture and Plantation Management, Wayamba University, Makandura, Sri Lanka

## Abstract

Skin disorders are one of the commonest conditions among school children in developing countries. There are only a few published studies available from Sri Lanka on the prevalence of skin disorders. A community-based cross-sectional study was carried out among five government-run schools randomly selected from the district of Gampaha, Sri Lanka, during 2016-2017. A total of 41 students between 5 and 16 years of age were randomly selected from each school. Sociodemographic profile and hygienic behaviors of selected students were assessed using a pretested interviewer-administered questionnaire. Students were examined by a medical officer for the presence of different skin disorders. The chi-squared test of association and binary logistic regression were used for the identification of the significance of socioeconomic factors and hygienic practices among the study participants. A total of 205 school children participated in the study. The commonest skin disorder was pediculosis (42.0%; *n*=86), followed by dandruff (8.3%; *n*=17), fungal infections (6.8%; *n*=14), and scabies (1.5%; *n*=3). Almost one-fourth of the study participants (22.9%; *n*=30/131) had more than one disorder, majored by *Pediculus captis* infestation with dandruff. Over one-third (36.1%; *n*=74) were free of any skin disorders. The prevalence of skin disorders was significantly high among females (87.3%; *n*=110), compared to males (26.6%; *n*=21). Presence of long hair, higher family size, and limited number of rooms in the house were risk factors associated with the prevalence of skin disorders. The commonest skin disorder was pediculosis, while scabies and fungal infections were scarce among school children in the district of Gampaha, Sri Lanka. Implementation of health education and monitoring programs at the school level for maintaining the dermal health status of school children is recommended.

## 1. Introduction

Ectoparasites and fungi are responsible for a heterogenous group of dermatoses, which are transmissible. Worldwide, scabies and pediculosis (capitis) are among the commonest ectoparasitic dermatoses that are present among children in developing countries, while pyodermas, dermatophytoses, and other fungal dermatoses too are reportedly of significance [1–3]. Scabies, which is caused by the human itch mite, *Sarcoptes scabiei*, was reported to have an average prevalence of 5–10% among children in endemic tropical regions [4]. Higher rates of infection ranging from 30–70% have been reported in the past from underprivileged communities living in India, South Pacific, and Northern Australia [5–7]. Scabies was added to the World Health Organization's (WHO) list of neglected tropical diseases in 2013 on account of its substantial morbidity resulting a higher burden of disease, which was mostly unrecognized [8]. Pediculosis caused by *Pediculus humanus capitis*, is another infection that is often reported among school-aged children, particularly girls [9]. Similarly, superficial fungal infections (SFIs) have been reported as a common cause of skin disease worldwide among school children [10].

Communities living in resource-poor settings in low-income countries have a higher burden of transmissible dermatoses, which is attributed to a variety of risk factors such as overcrowding, poor hygiene, lack of access to health care, inadequate treatment, malnutrition, and negligent social attitudes [11]. Many skin disorders such as scabies, pediculosis, pityriasis versicolor, acne vulgaris, and psoriasis can be rapidly diagnosed by their clinical features and need little or no further investigations. The management of skin disorders may range from simple reassurance to a variety of topical and systemic medications. It has been suggested that transmissible dermatoses among children are amenable to simple public health control measures, which could be incorporated into the school health programmes [12]. In communities with a high burden of transmissible dermatoses, interventions based on repeated mass or targeted treatment has been recommended [11]. Health education has also been documented as a useful tool for promotion of hygiene and health seeking behavior [10].

It is increasingly recognized that schools play an important role in inculcating healthy and hygienic habits among the younger generations as well as among the old, by take-home messages, which create awareness among the children, parents, or guardians on healthy living. Therefore, promoting personal cleanliness and environmental sanitation in schools may naturally safeguard children from transmissible dermatoses. More importantly, the negligent social attitudes of communities, which have affected the prevalence of common dermatoses such as pediculosis and scabies, could be reversed to some extent by counselling aimed at promoting the health seeking behavior of affected communities [10]. Such health educational messages could be imparted at schools and perhaps integrated with other public health control programs such as filariasis and intestinal helminthiases [13, 14].

School life lays the foundation for a better future and has a major impact on a host of other issues including health. Very often, formal education is the gateway to affluence and higher socioeconomic status. Thus, school education is compulsory in Sri Lanka and is provided free of charge by the state. The morbidity characteristics associated with dermatoses, specially the transmissible types, have been shown to constitute a serious setback to the education of a child [15, 16]. The visibility of lesions accompanied by constant scratching could lead to mental distress and lower self-esteem and affect the ability to adjust socially [11]. Thus, the presence of dermatoses among the younger inhabitants of resource-poor settings may lead to a vicious cycle and investment of scarce resources on education and may not deliver the desired results.

Despite the apparent burden of transmissible dermatoses such as scabies and pediculosis in other developing countries, little information is available regarding the prevalence of these infections and their sequelae among school children in Sri Lanka [17]. There are no published reports on national-level surveys of dermatoses among school children in Sri Lanka. Also, the risk factors for transmission of dermatoses among children have not been studied in Sri Lanka. Therefore, the present study was undertaken with the objective of determining the prevalence of superficial skin disorders and factors associated with their spread among children in the district of Gampaha, Sri Lanka.

## 2. Methodology

### 2.1. Study Design

**Figure 1:**
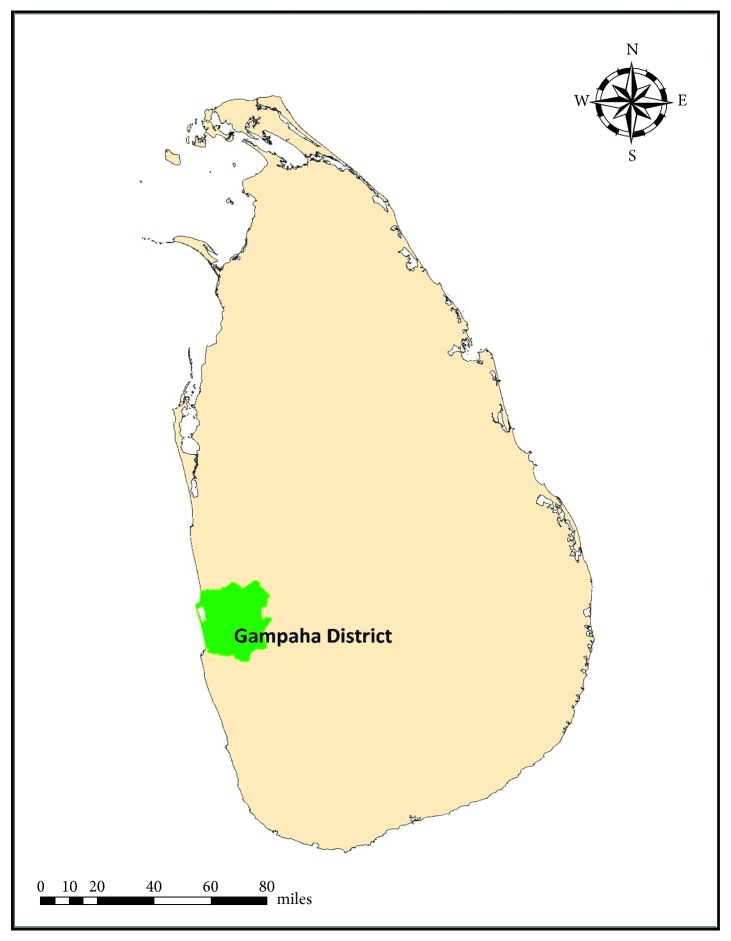
Location of the Gampaha District in Sri Lanka.

A community-based cross-sectional study was conducted among five randomly selected government-run schools (sampling units), in the district of Gampaha during the scholastic year 2016-2017. The district of Gampaha, situated in the Western Province of Sri Lanka (Figure 1), is the second most populous district, with 2.3 million inhabitants in a land area of 1,387 km^2^, and comprises urban, semiurban, and rural settings [18]. Based on the concept of cluster sampling, 5 schools were selected as the major sampling sites and the required sample size was calculated by taking the prevalence as 10% and absolute precision as 4% at a confidence level of 95%. A total of 41 students between 5 and 16 years of age were recruited from each school by following simple random sampling technique for the study to attain a sample size of 205. The study participants were assigned to one of the four age groups as follows: 5–7 years, 8–10 years, 11–13 years, and above 13 (>13) years of age.

### 2.2. Collection of Sociodemographic Information

Pretested questionnaires were used to assess the sociodemographic profiles and the hygienic behaviors of study participants. The sociodemographic section of the questionnaire that included age, gender, family size, number of household occupants, number of rooms in the house, parental education, occupation, and the average monthly household incomes was completed by mothers or principal caretakers, while hygienic behaviors of students were assessed directly by interviewers. The hygienic behaviors such as the frequency of hair washings, sharing of combs and brushes, and any recent infestation/infection events among family members were noted by direct interviews with study participants with the support of their guardian, where necessary. Details regarding previous treatments for skin infections among family members were also noted in the individual data sheets, based on the response of the guardian and the treatment cards issued for previous treatments for the family by the medical officers. All the questionnaires were filled via face-to-face interviews with the student and a guardian by a well-trained team of interviews (2 medical officers with MBBS qualification).

### 2.3. Clinical Examination for Skin Disorders

Students were clinically examined by a medical officer. Diagnosis of scabies was based on the following clinical criteria: skin lesions consisting of itchy rash (reddish papules/pustules less than 5 mm in diameter), which may or may not be accompanied by small raised flattened burrows on hands, interdigital spaces, and volar aspects of wrists and elbows [19]. The presence of other skin disorders, namely, impetigo (red sores on the face, nose, mouth, hands, or feet), atopic eczema (itchy, red, swollen, and cracked skin with clear fluid in affected areas), alopecia (bald spots on the scalp, each about the size of a coin), dandruff (flaking of the skin on scalp), and fungal infestations (red, irritated, or scaly rash), were also noted.

Hair and scalp area of all the participants were examined for head lice infestation, dandruff, and tinea infections using a hand lens. A student was considered infested with lice if at least one adult, nymph, or nit was present [20].

### 2.4. Statistical Analysis

All collected data were double-checked and verified on the same day for completeness and consistency. The data were then entered into Microsoft Access® data sheets (Version 2007), while adhering to quality controlling procedures by trained personnel. The accuracy of data was routinely checked by cross tabulations and logical checks. Discrepant data were checked against original data forms, and any mistakes were promptly corrected. The chi-squared test of association was used to identify the significance of variables (socioeconomic and behavioral factors) with skin disorders and parasitic and fungal infestations in the study population. The binary logistic regression was used to calculate the odds ratio (OR) along with 95% confidence intervals of the OR for each risk factor. All statistical analysis was done using the SPSS package 23.

## 3. Results

### 3.1. Prevalence of Different Skin Disorders and Associated Clinical Orientations

**Figure 2:**
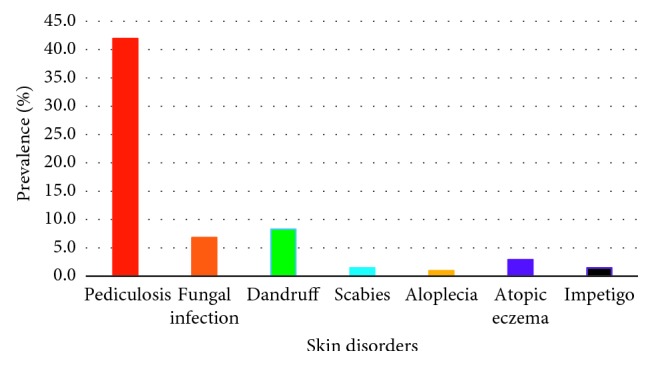
Percentage prevalence of different skin disorders among the sampled student population.

A total of 205 school children were enrolled for the current study. The overall prevalence of all skin disorders was 63.9% (*n*=131). The majority had pediculosis (42.0%; *n*=86), followed by dandruff (8.3%; *n*=17). Fungal infections consisting mostly of pityriasis or tinea versicolor were detected (6.8%; *n*=14), while scabies (1.5%; *n*=3) and impetigo (1.5%; *n*=3) had a lower prevalence. Alopecia was detected in a minority of 0.98% (*n*=2). Over one-third of the student population (36.1%; *n*=74) did not have any dermatoses (Figure 2), and nearly one-quarter of the surveyed population (22.9%; *n*=30/131) suffered from more than one skin disorder. The clinical manifestations associated with different skin disorders were observed to be different. Of them, scalp pruritus (63.4%; *n*=83) was detected as the predominant clinical feature, followed by scaly discolored patches in pityriasis (13.0%; *n*=17), skin rash (6.1%; *n*=8), and fever (2.3%; *n*=3).

### 3.2. Sociodemographic Risk Factors for the Prevalence of Skin Disorders

**Figure 3:**
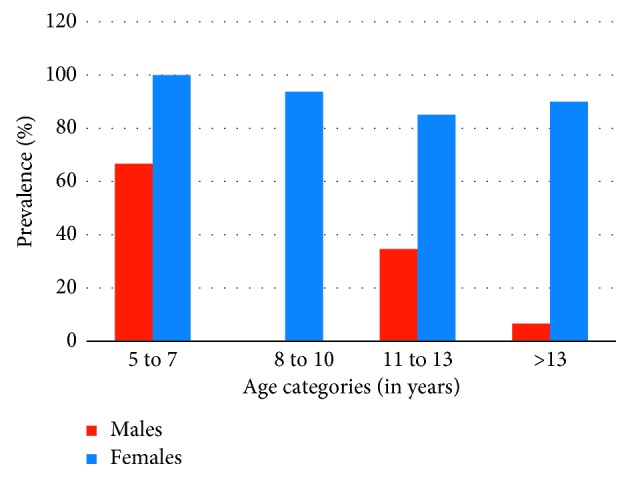
Percentage prevalence of skin disorders among male and female students belonging to different age groups, within the study population.

Sociodemographic characteristics of the study population, odds ratios (ORs), and 95% confidence intervals (CIs) of ORs for the prevalence of skin disorders are indicated in Table 1. Among the sampled population of children, females had a significantly higher (*P*=0.001; OR = 18.99 and CI = 16.48–19.71) prevalence rate of skin disorders (87.3%; *n*=110), compared to the prevalence among male students (26.6%; *n*=21). The highest prevalence was observed among the students belonging to the age group of 5–7 years (83.3%; OR = 1), followed by 11–13 (66.2%; OR = 0.39; CI = 0.22–0.52) and 8–10 years (60%; OR = 0.30; CI = 0.19–0.59). As depicted by the age specific prevalence rates and OR, the younger age category of 5–7 years showed the highest susceptibility to skin disorders (Figure 3). Meanwhile, statistics of the chi-squared test of association denoted that the association between the different age categories and the prevalence of skin disorders is not significant (*P*=0.209).

The association between the education level of the father and the prevalence of skin disorders remained not significant (*P*=0.414), while the maternal education showed a statistically significant association (*P*=0.041). However, as indicated by the OR, children having fathers with an education level up to primary education had a relatively higher odds for the prevalence of skin disorders (OR = 2.05; CI = 1.01–2.74), which was not statistically significant (*P*=0.414). In case of maternal education, children of mothers with higher educational levels had greater occurrence of skin disorders. Mothers with higher educational tiers such as secondary education completed (OR = 5.41; CI = 3.97–7.14) and graduates and diploma holders (OR = 6.00; CI = 4.47–8.83) were characterized with higher odds for prevalence of skin disorders among children.

The majority of the study participants (82%; *n*=168) represented the average Sri Lankan family size of 4–6 members in the family. The highest prevalence rate of skin disorders was observed among the families with >6 members (88.3%; OR = 13.93; CI = 11.69–15.67), followed by 4–6 members (64.9%; OR = 3.43; CI = 2.38–4.40). Therefore, the results of the current study suggest that a larger family size significantly contribute towards a higher prevalence of skin disorders among children.

### 3.3. Impact of Housing Characteristics and Behavioral Practices on Skin Disorders

Students residing in houses with fewer number of rooms (1–3) denoted a higher prevalence rate of skin disorders (70.3%; *n*=102). Furthermore, single occupancy of a bedroom had the lowest infestation rate (28.6%). The highest prevalence of skin disorders was observed among students sharing the bedroom with >4 family members (93.7%; OR = 18.50; CI = 14.41–20.68), followed by 3–4 members (86.3%; OR = 15.71; CI = 13.37–16.86). Therefore, the overcrowding nature of rooms (presence of a low number of rooms and occupancy of a room by a high number of members) could be recognized as a risk factor associated with the higher prevalence rates of skin disorders among the study participants. Furthermore, the association between the prevalence of skin disorders and the number of rooms in the household and the number of occupants sharing a room was significant (*P*=0.048 and *P*=0.032) with notable ORs, as indicated in Table 2.

### 3.4. Association of Behavioral Practices with the Prevalence of Pediculosis

Being the most abundant skin infestation among the studied student population, association of practices of students on the incidence of pediculosis was specially investigated. Among 86 students with pediculosis, majority were girls (87.2%; OR = 9.09; CI = 7.21–9.82), while only 11 males were infested. Females with long hair (length of the hair beyond the neck) had relatively a higher prevalence (70.6%; *n*=72). In the case of male students, 26.9% (*n*=7) with hair longer than 3 cm were infested (Table 3).

The findings of the current study suggested that the presence of long hair was a significant risk factor associated with high odds ratio for pediculosis prevalence in both males (*P*=0.046; OR = 9.58; CI = 7.66–11.76) and females with long hair (*P*=0.039; OR = 16.80; CI = 14.40–18.08). Frequency of bathing, hair washing, or shampooing had no significant association to the prevalence of pediculosis, as indicated by the results of the chi-squared test of association (Table 3).

It was rather interesting to note that only 27.6% (*n*=8) of the students were infested with lice, among 29 students that did not use any shampoo during bathing, while students who practiced shampooing denoted notable infestation rates (Table 3). Comparatively lower proportion of students having a high number of hair washes such as 2 (OR = 0.69; CI = 0.49–0.79) and >2 (OR = 0.72; CI = 0.61–0.91) were reported with a lower odds ratio of pediculosis prevalence, which was not statistically significant (*P*=0.328). Students who shared hair grooming appliances such as combs and hairbrushes with other family members had a significantly higher prevalence of pediculosis (OR = 6.63; CI = 5.02–7.30). Furthermore, the students that were not using a louse comb had a relatively higher chance for the prevalence of pediculosis (OR = 1.38; CI = 0.95–1.68), which was not significant.

Overall, larger family size, overcrowding of rooms, presence of long hair, type of comb, and sharing of combs were identified as significant risk factors for skin disorders among school children.

## 4. Discussion

Several attempts have been made in other developing countries in the past to determine the prevalence rate of skin disorders among school children [21, 22]. Over two decades ago, the prevalence rates of skin diseases among school children was documented to range between 21 and 87% by the World Health Organization (WHO) [23]. The prevalence of infectious dermatoses is an index of socioeconomic development [24]. Therefore, the highest burdens of transmissible dermatoses occur in resource-poor communities in developing countries. Information on the prevalence of skin infections among Sri Lankan school children was limited. The high rates of pediculosis and scabies (10% and 25%, respectively) reported among children living with their prison-inmate mothers in 1999 [17] were not surprising as overcrowding in such institutions coupled with deficient hygiene facilitates the spread of infections. Surveillance done among a suburban community in Sri Lanka in year 2000 has reported that almost half the population had skin disorders (47.6%, *n*=1806). Fungal infections followed by dermatitis have been identified as commonest problems [25]. Therefore, determination of the prevalence of skin diseases in Sri Lanka was a timely requirement.

The current study reports that dermatoses were common among school children in the district of Gampaha (overall prevalence, 63.9%), and the commonest types identified in the descending order were pediculosis, dandruff, fungal infections, and scabies. The predominant of them, pediculosis due to *Pediculus captis* infestation, was more common among girls than boys. This may be due to several factors such as girls are having longer hair in comparison with boys, frequent close head contact between girls, and perhaps a higher probability of sharing hair grooming appliances amongst them, due to the heightened grooming and combing requirements that accompany longer hair [26, 27].

It is important to note that even though the spectrum of diseases was fewer in the present study, the prevalence rate for any infection was comparatively higher compared to some studies conducted in other developing countries such as Nigeria and India, where prevalence rates of 39.6% and 29.5%, respectively, have been reported among school children, [3, 28]. Many of these disorder types were infective dermatoses with superficial fungal infections (dermatophytoses and pityriasis versicolor, scabies, pediculosis, and acne vulgaris infection) among the children [3, 28].

The occurrence of fungal infections and dandruff in the present study may be due to the warm humid climate in the region that leads to accumulation of sweat, facilitating the growth of fungi, which predispose to scalp dandruff [25]. In the present study, there were none with tinea capitis infection, which reportedly has a low incidence in Sri Lanka attributed to the custom of having frequent head baths with soap and water [29]. It is of importance to note that the prevalence of scabies among this community was low. Several countries have also reported a reduction in the prevalence of scabies [25,30–32]. Improvements in socioeconomic status and living standards among communities have been mentioned as probable factors responsible for such a tendency [22, 30].

According to Perera et al., almost half of the studied population (50%) was affected with dermatoses and did not think it was necessary to seek treatment [25]. Similarly, in the current study, majority of the school children have not had any form of treatment for their dermatoses despite routine school medical inspections and access to free health care. Several studies have highlighted the deficient behavior in seeking health care among school children [22, 33]. This negligent attitude could be attributed to the ubiquitous nature of dermatoses and the assumption that skin diseases are not important since they do not constitute any metabolic disturbances [22]. However, primary and secondary morbidity of dermatoses may have a substantial impact on health [22]. Therefore, it is important to create awareness of the consequences of common skin disorders by health education and motivate children and their caretakers to obtain treatment.

Diagnosis of dermatoses is relatively easy and can be done by the affected individuals or their caretakers themselves. They can be effectively treated by topically applicable insecticides/acaricides or antifungals. In the case of scabies and pediculosis, all members of the household/institution should be simultaneously treated and followed up for a week by repeat applications. Since most of these skin infections are intricately linked to poverty, it is unlikely that they may be eradicated by medications alone; but a reduction in prevalence and intensity of skin disorders will presumably lead to a reduction in morbidity.

Parental education is assumed to play a major role in preventing contagious diseases. However, some studies have indicated that educational levels of parents were not significantly associated with skin disorders [34–36]. In the present study, the prevalence of skin infections was higher among children having mothers with a higher level of education. The probable reason for the above outcome was provided by analyzing the maternal employment status. The lowest prevalence of skin infections was among children of unemployed (stay-at-home) mothers, who naturally had more time to attend to the health requirements of their offspring.

Children living in houses with a lesser number of rooms (1–3) had significantly higher rates of skin disorders, indicating that overcrowding was a significant risk factor. In agreement with other studies, a larger family size (>6 members) was also identified as a significant risk factor for skin infestations [26, 37, 38]. Students of larger families were probably at a higher risk of infection, due to overcrowding, frequent exposure to infections through siblings, and inadequate parental care and supervision [39, 40].

Sharing of personal items such as towels and combs at home has been identified to be significant in the spread of skin disorders among school children [22, 39, 41]. The present study revealed that sharing of combs within the family was a significant risk factor for pediculosis. This study had several limitations. The size of the study population was small and was over-represented by children of the age category 11–13 years probably due to sampling errors and the longer school sessions of older students increasing their availability to participate in the survey. Skin infections on unexposed body areas may have been missed due to the inability to conduct a proper general examination within the school environment.

Based on the current study, we cannot generalize on the status of skin disorders in Sri Lanka as the study was confined to a single district. Furthermore, it has some methodical limitations such as small sample size. In addition, the study was conducted among school students between 5 and 16 years of age. Therefore, some participants may be too young to provide accurate data about their parents and sociodemographic information. On the other hand, diagnosis was entirely clinical and did not use a microscope or skins scraping and no further validation was conducted for confirmation of fungal infections. As prevalence and types of skin diseases are known to vary according to socioeconomic status, climatic regions, and religious customs [42, 43], further studies in other parts of the country to assess the burden of dermatoses are recommended. This study warrants the need for reinforcing health education at schools and perhaps inspection and supervision of students by school authorities for common dermatoses. Parental motivation to focus attention on the health status of children is also emphasized.

## 5. Conclusion

Pediculosis was identified as the most prevalent superficial dermatoses among school children in the Gampaha District. Female gender, having long hair, maternal employment status, overcrowding nature of family members, and common usage of hair grooming appliances were recognized as significant risk factors associated with skin disorders. Scabies and fungal dermatoses were scarce among the studied population. Implementation of health education and monitoring programs at the school level for maintaining dermal health status of school children is recommended.

## Figures and Tables

**Table 1:** Basic socio-demographic factors of the study population.

Variable	Total respondents		Prevalence of skin disorders		*P* value	Odds ratio	95% confidence interval for the odds ratio
	Number	Percentage (%)	Number	Percentage (%)			
Gender							
Male	79	38.5	21	26.6	0.001	1.00	
Female	126	61.5	110	87.3		18.99	16.48–19.71

Age							
5–7	6	2.9	5	83.3	0.209	1.00	
8–10	25	12.2	15	60.0		0.30	0.19–0.59
11–13	139	67.8	92	66.2		0.39	0.22–0.52
>13	35	17.1	19	54.3		0.24	0.17–0.41

Fathers education							
Incomplete primary education	8	3.9	4	50.0	0.414	1.00	
Primary education completed	61	29.8	41	67.2		2.05	1.01–2.74
Secondary	131	63.9	83	63.4		1.73	1.26–3.16
University/diploma or similar	5	2.4	3	60.0		1.50	0.95–1.96

Mothers education							
Incomplete primary education	6	2.9	2	33.3	0.041	1.00	
Primary education completed	58	28.3	26	44.8		1.63	1.21–3.40
Secondary	137	66.8	100	73.0		5.41	3.97–7.14
University/diploma or similar	4	1.5	3	75.0		6.00	4.47–8.83

Fathers occupation							
Government servant	31	15.1	24	77.4	0.243	1.00	
Self-employment	18	8.8	10	55.6		0.36	0.22–0.62
Traders/business	8	3.4	7	87.5		2.04	1.43–4.30
Dead/disabled	5	2.4	4	80.0		1.17	1.04–2.51
Labourer	133	65.4	84	63.2		0.50	0.35–0.89
Other	10	4.9	2	20.0		0.07	0.01–0.15

Mothers occupation							
Housewife	126	61.5	63	50.0	0.087	1.00	
Government servant	5	2.4	4	80.0		4.00	2.82–6.22
Labourer	19	9.3	16	84.2		5.33	3.91–6.61
Self-employment	5	2.4	4	80.0		4.00	2.82–6.22
Traders/business	16	7.8	13	81.3		4.33	3.09–5.64
Other	34	16.6	31	91.2		10.33	8.35–11.57

Family size							
1 to 3	20	9.8	7	35.0	0.034	1.00	
4 to 6	168	82.0	109	64.9		3.43	2.38–4.40
>6	17	8.2	15	88.3		13.93	11.69–15.67

**Table 2:** Practices of the study population with respect to incidence of different skin disorders.

Variable	Total respondents		Prevalence of skin disorders		*P* value	Odds ratio	95% confidence interval for the odds ratio number
	Number	Percentage (%)	Number	Percentage (%)			
No of rooms							
1 to 3	145	70.7	102	70.3	0.048	1.00	
4 to 5	50	24.4	27	54.0		0.49	0.31–0.64
>6	10	4.9	2	20.0		0.11	0.05–0.25

No of people living in the same room							
1	28	13.7	8	28.6	0.032	1.00	
2	110	53.7	64	58.2		3.48	2.42–4.38
3 to 4	51	24.9	44	86.3		15.71	13.37–16.86
>4	16	7.8	15	93.8		18.50	14.41–20.68

Bathing facilities in the house							
Bathroom	74	36.1	43	58.1	0.351	1.00	
Well	36	17.6	22	61.1		1.13	0.97–1.95
River	2	1.0	1	50.0		0.72	0.58–1.05
Tap line	72	35.1	51	70.8		1.75	1.27–2.44
Cement tank	4	2.0	3	75.0		2.16	1.51–4.47
More than one	17	8.3	11	64.7		1.32	0.95–1.52

Sharing towel							
Sharing towel	33	16.1	28	84.9	0.242	1.00	
Not sharing towel	172	83.9	103	59.9		0.27	0.19–0.39

**Table 3:** Practices of the study population with respect to incidence of pediculosis.

Variable	Total respondents		Prevalence of skin disorders		*P* value	Odds ratio number	95% confidence interval for the odds ratio
	Number	Percentage (%)	Number	Percentage (%)			
Gender							
Male	79	38.5	11	14.0	0.001	1.00	
Female	126	61.5	75	59.5		9.09	7.21–9.82

Hair length of females							
Short	24	11.7	3	12.5	0.039	1.00	
Long	102	49.8	72	70.6		16.80	14.40–18.08

Hair length of males							
<2 cm	27	13.2	1	3.7	0.046	1.00	
3 cm	26	12.7	3	11.5		3.39	2.35–5.72
>3 cm	26	12.7	7	26.9		9.58	7.66–11.76

No of hair washes per week							
1	8	3.9	4	50.0	0.328	1.00	
2	22	10.7	9	41.0		0.69	0.49–0.79
>2	175	85.4	73	41.7		0.72	0.61–0.91

Shampooing per week							
1	39	19.0	19	48.7	0.271	1.00	
2	39	19.0	17	43.6		0.27	0.17–0.47
>2	98	47.8	42	42.9		0.26	0.18–0.38
Not using shampoo	29	14.2	8	27.6		0.21	0.14–0.45

Combing							
Using louse comb	55	26.8	20	36.4	0.065	1.00	
Not using louse comb	150	73.2	66	44.0		1.38	0.95–1.68

Sharing items							
Not sharing comb	81	39.5	14	17.3	0.028	1.00	
Sharing comb	124	60.5	72	58.1		6.63	5.02–7.30

## Data Availability

The data used to support the findings of this study are available from the corresponding author upon request.

## References

[1] Komba E. V., Mgonda Y. M. (2010). The spectrum of dermatological disorders among primary school children in Dares Salaam. *BMC Public Health*.

[2] Inanir I., Sahin M. T., Gunduz K., Dinc G., Turel A., Ozturkcan S. (2002). Prevalence of skin conditions in primary school children in Turkey: differences based on socioeconomic factors. *Pediatric Dermatology*.

[3] Ogunbiyi A. O., Owoaje E., Ndahi A. (2005). Prevalence of skin disorders in school children in ibadan, Nigeria. *Pediatric Dermatology*.

[4] World Health Organization (2005). *Epidemiology and Management of Common Skin Diseases in Children in Developing Countries*.

[5] Nair B. K., Joseph A., Kandamuthan M. (1977). Epidemic scabies. *Indian Journal of Medical Research*.

[6] Carapentis J. R., Connors C., Yarmirr D., Krause V., Currie B. J. (1997). Success of scabies control program in an Australian aboriginal community. *Pediatric Infectious Disease Journal*.

[7] Currie B. J., Connors C. M., Krause V. L. (1994). Scabies programs in aboriginal communities. *Medical Journal of Australia*.

[8] Engelman D., Kiang K., Chosidow O. (2013). Toward the global control of human scabies: introducing the international alliance for the control of scabies. *PLoS Neglected Tropical Diseases*.

[9] Maguire J. H., Spielman A. (1998). Ectoparasite infestations and arthropod bites and stings. *Harrisons Principles of internal Medicine*.

[10] Olutoyin O. O., Onayemi O., Gabriel A. O. (2017). Risk factors associated with acquiring superficial fungal infections in school children in South Western Nigeria: a comparative study. *African Health Sciences*.

[11] Feldmeier H., Heukelbach J. (2009). Epidermal parasitic skin diseases: a neglected category of poverty-associated plagues. *Bulletin of the World Health Organization*.

[12] Kalu E. I., Wagbatsoma V., Ogbaini-Emoven E., Nwadike V. U., Ojide C. K. (2015). Age and sex prevalence of infectious dermatoses among primary school children in a rural South-Eastern Nigerian community. *Pan African Medical Journal*.

[13] Taplin D., Meinking T. L., Porcelain S. L. (1991). Community control of scabies: a model based on use of permethrin cream. *The Lancet*.

[14] Heukelbach J., Winter B., Wilcke T. (2004). Selective mass treatment with ivermectin to control intestinal helminthiases and parasitic skin diseases in a severely affected population. *Bull World Health Organ*.

[15] Kingman S. (2005). Growing awareness of skin diseases. *Bull World Health Organ*.

[16] Wagbatsoma V. A., Okojie O. H. (2004). Psychological effects of river blindness in a rural community in Nigeria. *Journal of the Royal Society for the Promotion of Health*.

[17] Senanayake M. P., Arachchi J. K., Wickremasinghe V. P. (2001). Children of imprisoned mothers. *Ceylon Medical Journal*.

[18] Department of Census and Statistics Sri Lanka, Statistics Department, 2012, http://www.statistics.gov.lk/

[19] Romani L., Koroivueta J., Steer A. C. (2015). Scabies and impetigo prevalence and risk factors in Fiji: a national survey. *PLoS Neglected Tropical Diseases*.

[20] Morsy T. A., Morsy A., Farrag A. M., Sabry A. H., Salama M. M., Arafa M. A. (1991). Ecto and endoparasites in 2 primary schools in Qualyob. *Journal of the Egyptian Society of Parasitology*.

[21] Fiqueroa J. L., Fuller L. C., Abraha A., Hay R. J. (1996). The prevalence of skin disease among school children in rural Ethiopia-a preliminary assessment of dermatologic needs. *Pediatric Dermatology*.

[22] Amoran O. E., Runsewe-Abiodun O. O., Mautin A. O., Amoran I. O. (2011). Determination of dermatological disorders among school children in Sagaumu, Nigeria. *Educational Research*.

[23] WHO (1997). *Improving Child Health, Integrated Management of Childhood Illnesses: the Integrated Approach*.

[24] Olasode O. A., Henshaw E. B., Akpan N. A., Otu A. A. (2010). Cutaneous infections in patients presenting in a skin clinic in the tropics. *International Journal of Tropical Medicine*.

[25] Perera A., Atukorale D. N., Sivayogan S., Ariyaratne V. S., Karunaratne L. A. (2000). Prevalence of skin diseases in suburban Sri Lanka. *Ceylon Medical Journal*.

[26] Alzain B. (2012). *Pediculosis captis* infestation in school children of a low socioeconomic area of the North Gaza Governorate. *Turkish Journal of Medical Sciences*.

[27] Jinadu M. K. (1985). Pediculosis humanus capitis among primary school children in lie-ife, Nigeria. *Journal of the Royal Society of Health*.

[28] Kumar A. S., Devi B. N., Jahnavi K., Varma P. (2016). A study on prevalence of skin infections among School children in Hyderabad, Telangana state. *International Journal of Contemporary Medical Research*.

[29] Atapattu M. C. (1989). A study of tinea capitis in Sri Lanka. *Journal of Medical and Veterinary Mycology*.

[30] Terry B., Kanjah F., Sahr F., Kortequee S., Dukulay I., Gbakima A. A. (2001). Sarcoptes scabiei infestation among children in a displacement camp in Sierra Leone. *Public Health*.

[31] Sharma V., Silverberg N. B., Howard R., Tran C. T., Laude T. A., Frieden I. J. (2001). Do hair care practices affect the acquisition of tinea capitis?. *Archives of Pediatrics & Adolescent Medicine*.

[32] Ciftci I. H., Karaca S., Dogru O., Cetinkaya Z., Kulac M. (2006). Prevalence of pediculosis and scabies in preschool nursery children of Afyon, Turkey. *Korean Journal of Parasitology*.

[33] Mgonda Y. M., Lutale J. J. (2001). Acne in secondary school students in Dares Salaam: perceptions, attitudes and implications. *Tanzania Medical Journal*.

[34] Silverio A. D., Zeccara C., Serra F., Ubezio S., Mosca M. (1995). Pityriasis versicolor in a newborn Pityriasis versicolor bei einem Neugeborenen. *Mycoses*.

[35] Silva V., Di Tilia C., Fischman O. (1995). Skin colonization by Malassezia furfur in healthy children up to 15 years old. *Mycopathologia*.

[36] Akpata L. E., Gugnani H. C., Utsalo S. J. (1990). Pityriasis versicolor in school children in cross River state of Nigeria. *Mycoses*.

[37] El-Rifaie A. A., Meabed M. H., Mostafa O. A. (2000). Epidemiology of scabies and pediculosis capitis among primary school children in Beni Suef Governorate. Egypt. *International Journal of Medical Sciences*.

[38] El-Shafie O., El-Shazly H. (2000). Head lice among primary school children in Minofiya and the effect of different protocols of treatment. *Egyptian Journal of Medical Sciences*.

[39] Willems S., Lapeere H., Haedens N., Pasteels I., Naeyaert J. M., De Maeseneer J. (2005). The importance of socio-economic status and individual characteristics on the prevalence of head lice children. *European Journal of Dermatology*.

[40] Wegner Z., Racewicz M., Stanczak J. (1994). Occurrence of pediculosis capitis in a population of children from Gdansk, Sopot, Gdynia and the vicinities. *Applied Parasitol*.

[41] Heukelbach J., Feldmeier H. (2006). Scabies. *The Lancet*.

[42] Green M. S. (1989). Epidemiology of scabies. *Epidemiologic Reviews*.

[43] Kannathasan S., Murugananthan A., Rajeshkannan N., de Silva N. R. (2012). Cutaneous larva migrans among devotees of the nallur temple in jaffna, Sri Lanka. *PLoS One*.

